# Supplementary material for: Specific fibroblast subpopulations and neuronal structures provide local sources of Vegfc-processing components during zebrafish lymphangiogenesis
Source: Nat Commun. 2020 Jun 1;11:2724. doi: 10.1038/s41467-020-16552-7 (PMC7264274; doi:10.1038/s41467-020-16552-7)
Supplement: Supplementary file 1 — Supplementary Information [file 41467_2020_16552_MOESM1_ESM.pdf]

Supplementary Information

Specific fibroblast subpopulations and neuronal structures provide local sources of Vegfc-processing components during zebrafish lymphangiogenesis

(Wang et al.)

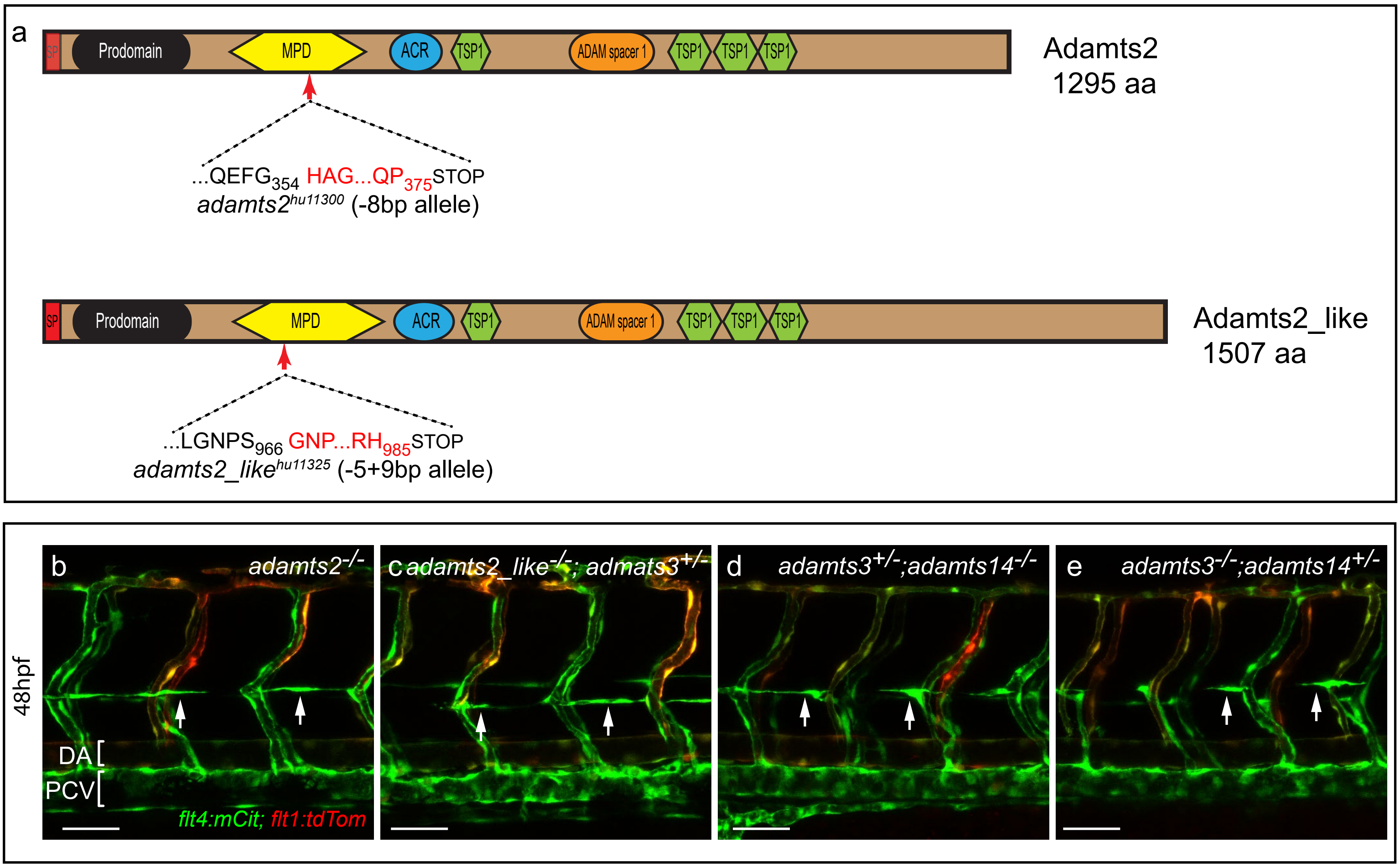

Supplementary Figure 1 Lymphangiogenesis is unaffected in *adamts2* or *adamts2_like* mutants and in embryos with one remaining wild-type allele of *adamts3*;*adamts14*.

a) An 8bp deletion in the MPD domain of *Adamts2* results in a frame-shift (red letters) after amino acid position 354 and a predicted premature stop codon after 375 amino acids. For Adamts2_like, targeting the MPD coding sequence resulted in an indel after amino acid position 966 and a predicted premature stop codon after 985 amino acids. SP: signal peptide, MPD: Metallopeptidase domain, ACR: ADAM cysteine-rich domain 2, TSP1: Thrombospondin type-1. b-e) *flt4:mCitrine; flt1:tdTomato* double transgenic embryos at 48hpf highlighting arterial ECs in red and venous and lymphatic structures in green. The formation of PLs is not disturbed in *adamts2* (b) or *adamts2_like* (c) homozygous mutants. d-e) Embryos with allelic combinations of *adamts3* and *adamts14* harboring at least one wild type copy of either gene show normal PL formation. Arrows highlight PL cells. Scale bars: 50 µm. EC: endothelial cells, DA: dorsal aorta, PCV: posterior cardinal vein, PL: parachordal lymphangioblast.

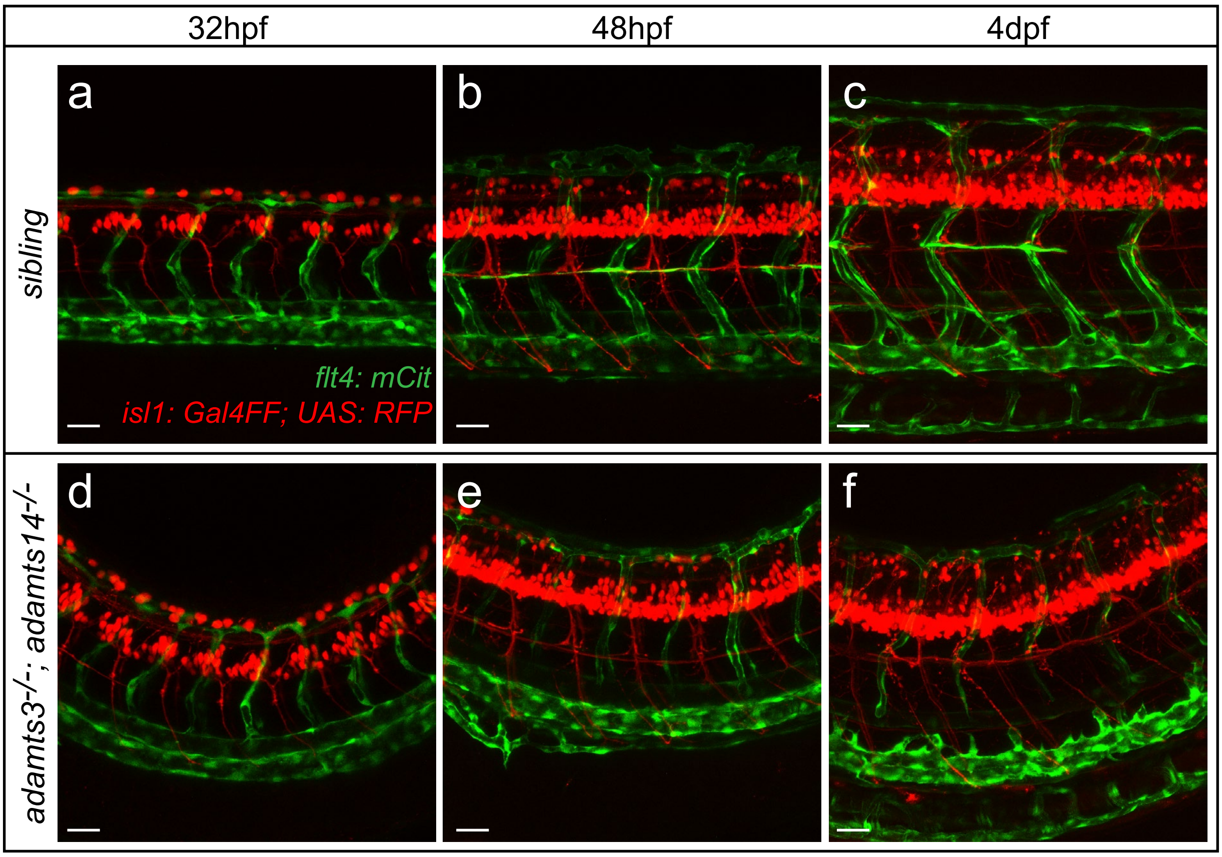

Supplementary Figure 2 The axonal pattern of motoneurons is not affected in *adamts3; adamts14* double mutants.

Motoneurons are visualized by *isl1:Gal4FF; UAS:RFP* in red, while venous and lymphatic structures are highlighted by *flt4:mCitrine* in green. a-f) Motoneuron morphology of sibling and *adamts3; adamts14* mutants at 32hpf, 48hpf and 4dpf. Scale bars: 50µm. hpf: hours post fertilization, dpf: days post fertilization.

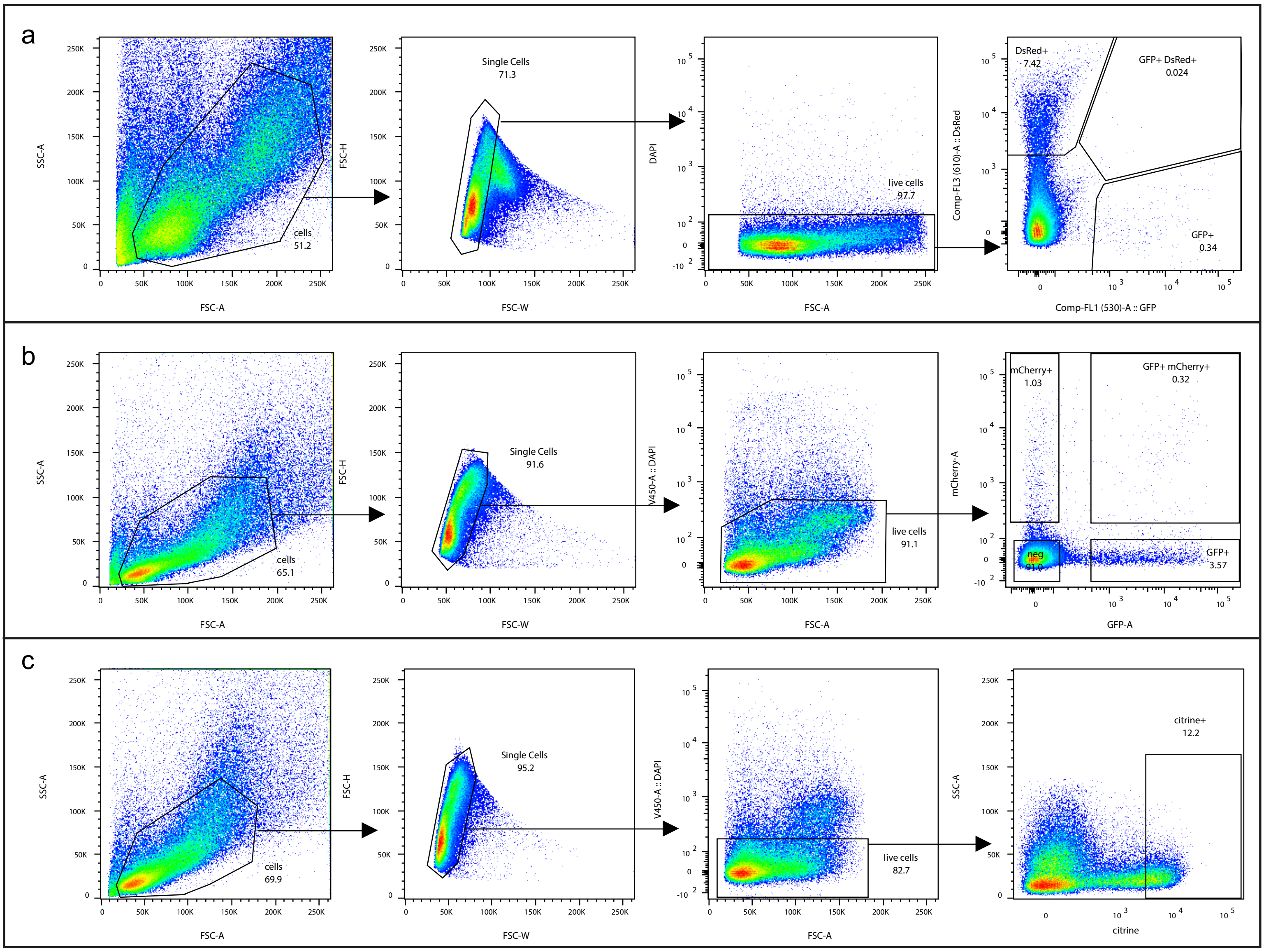

Supplementary Figure 3 FACS gating strategy.

a-c) Gating strategies for the FAC-sort of cells derived from *adamts3:Gal4FF; UAS:GFP; NBT:dsRed* (a), *vegfc:Gal4FF; UAS:GFP; kdrl:mCherry* (b) and *pdgfra:mCitrine* (c) transgenic embryos.

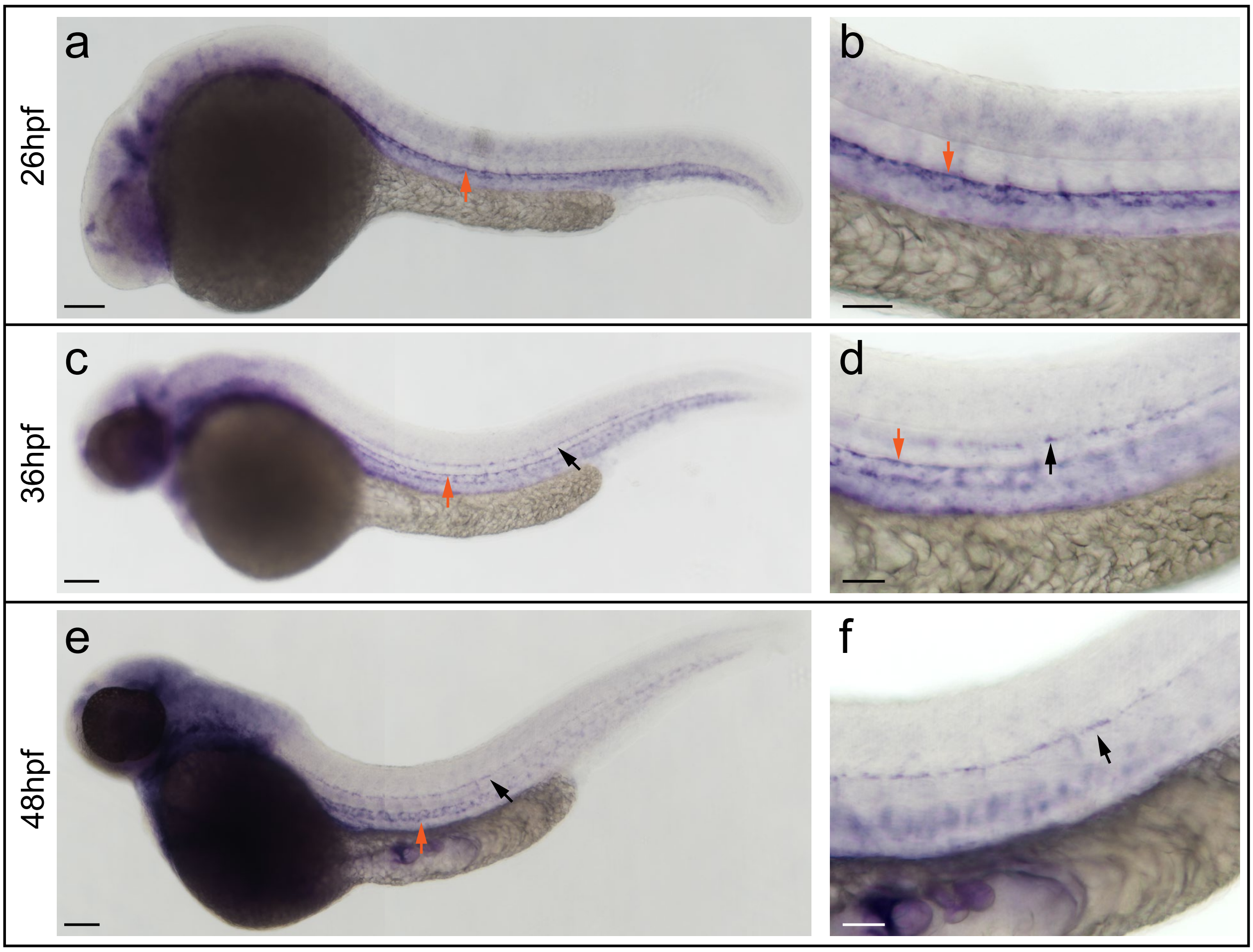

Supplementary Figure 4 *In situ* hybridization against *vegfc* using a milder proteinaseK treatment reveals expression at the horizontal myoseptum.

a, b) *vegfc* transcripts were detected in the hypochord, dorsal aorta (arrow) and in developing intersegmental arteries at 26hpf. b) Higher magnification of the trunk region with prominent staining of the hypochord (arrow). c, d) At 36hpf *vegfc* transcripts were detected in cells at the HM (black arrow), the hypochord and the dorsal aorta (orange arrow). d) Higher magnification of the trunk region. e, f) Expression of *vegfc* within the DA and in cells at the HM persist at 48hpf. f) High magnification of the trunk focusing at the HM level. Scale bars in a, c, e: 100µm; b, d, f: 50µm. hpf: hours post fertilization, DA: dorsal aorta, HM: horizontal myoseptum.

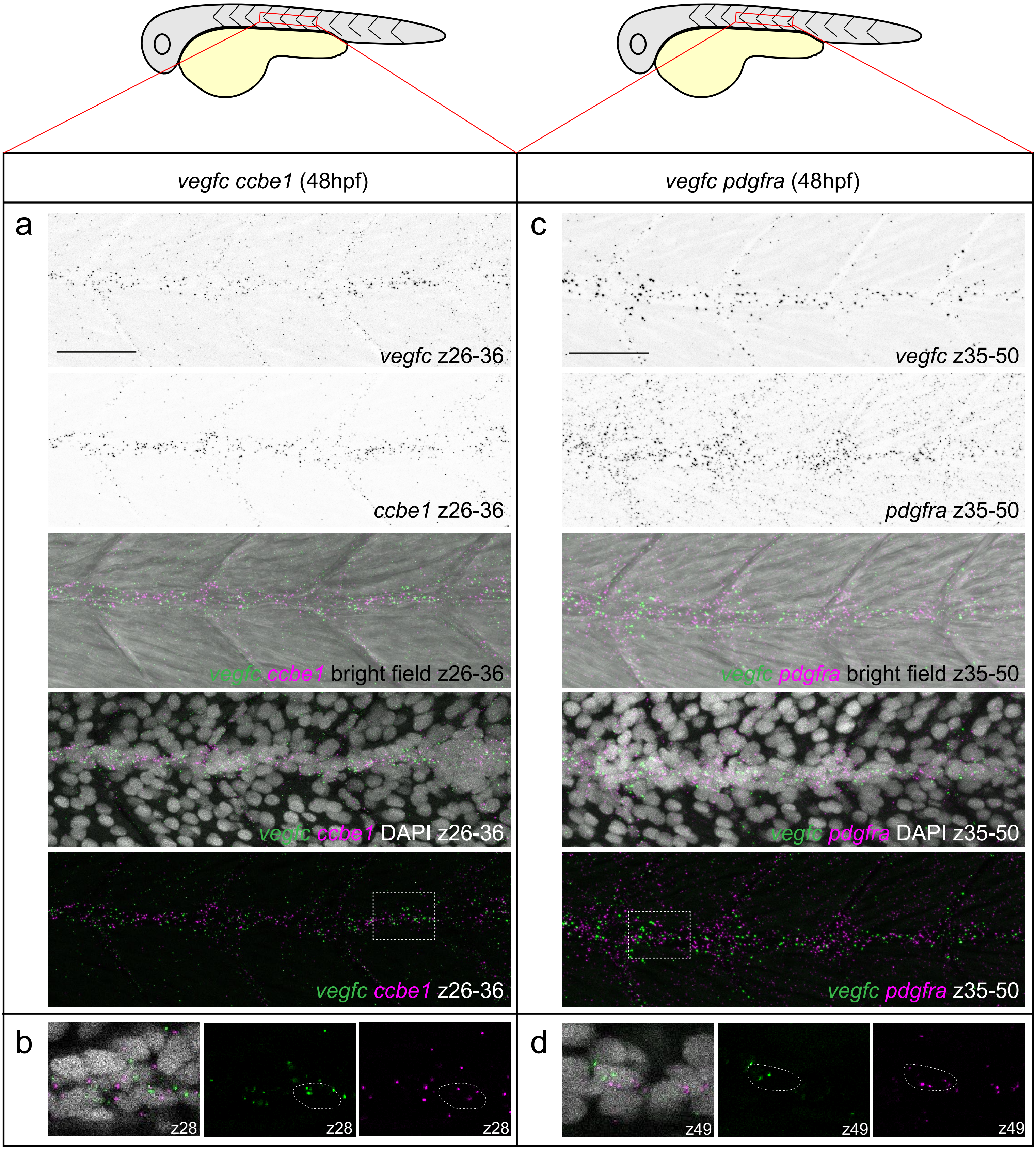

Supplementary Figure 5 Co-expression of *vegfc* with *ccbe1* and *pdgfra* mRNA in cells at the HM as detected by pairwise RNAscope analysis.

Simultaneous detection of mRNA granules for *vegfc* and *ccbe1* (a, b), *vegfc* and *pdgfra* (c,d) in embryos at 48hpf. Overview pictures of the HM region (a, c) represent partial z-projections of the indicated ranges and depict the distribution of the respective mRNA granules and the tight packing of nuclei (DAPI staining) in this area. Co-expression of individual transcript combinations was assessed on single confocal sections (b, d) focusing exclusively on RNA granules that reside within the same nucleus (indicated by DAPI staining or outlined by a dotted circle). Scale bars: 50µm. hpf: hours post fertilization, HM: horizontal myoseptum.

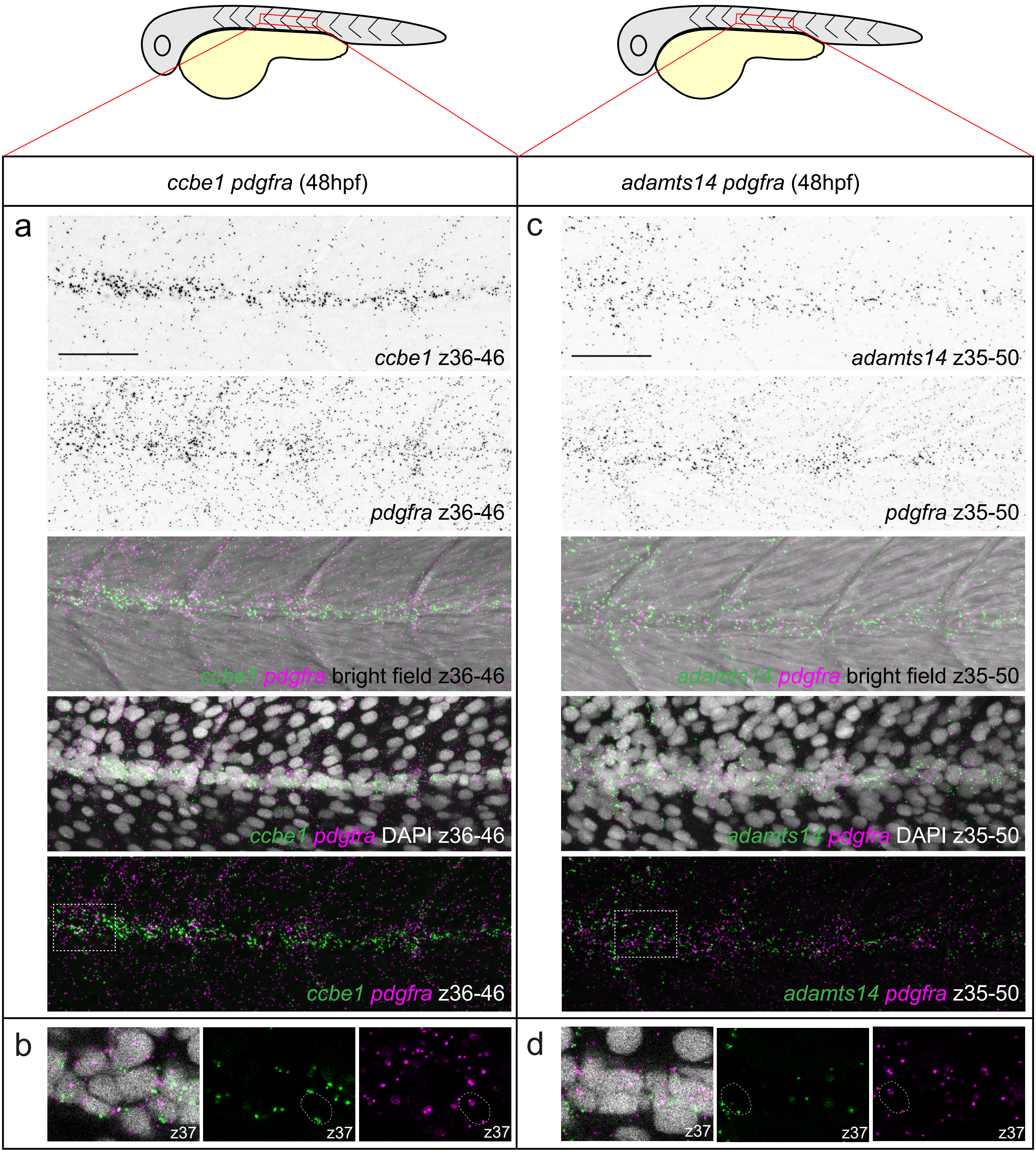

Supplementary Figure 6 Pairwise RNAscope analysis of *pdgfra* with *ccbe1* and *adamts14* transcripts reveals co-expression in cells at the HM.

Detection of mRNA granules for *ccbe1* and *pdgfra* (a, b), *adamts14* and *pdgfra* (c, d). The overview pictures of the HM region (a, c) represent partial z-projections of the indicated ranges and show the distribution of the respective mRNA granules. Co-expression of both transcript combinations was assessed on single confocal sections (b, d) taking only RNA granules into account that reside within the same nucleus (indicated by DAPI staining or outlined by a dotted circle). Scale bars: 50µm. hpf: hours post fertilization, HM: horizontal myoseptum.

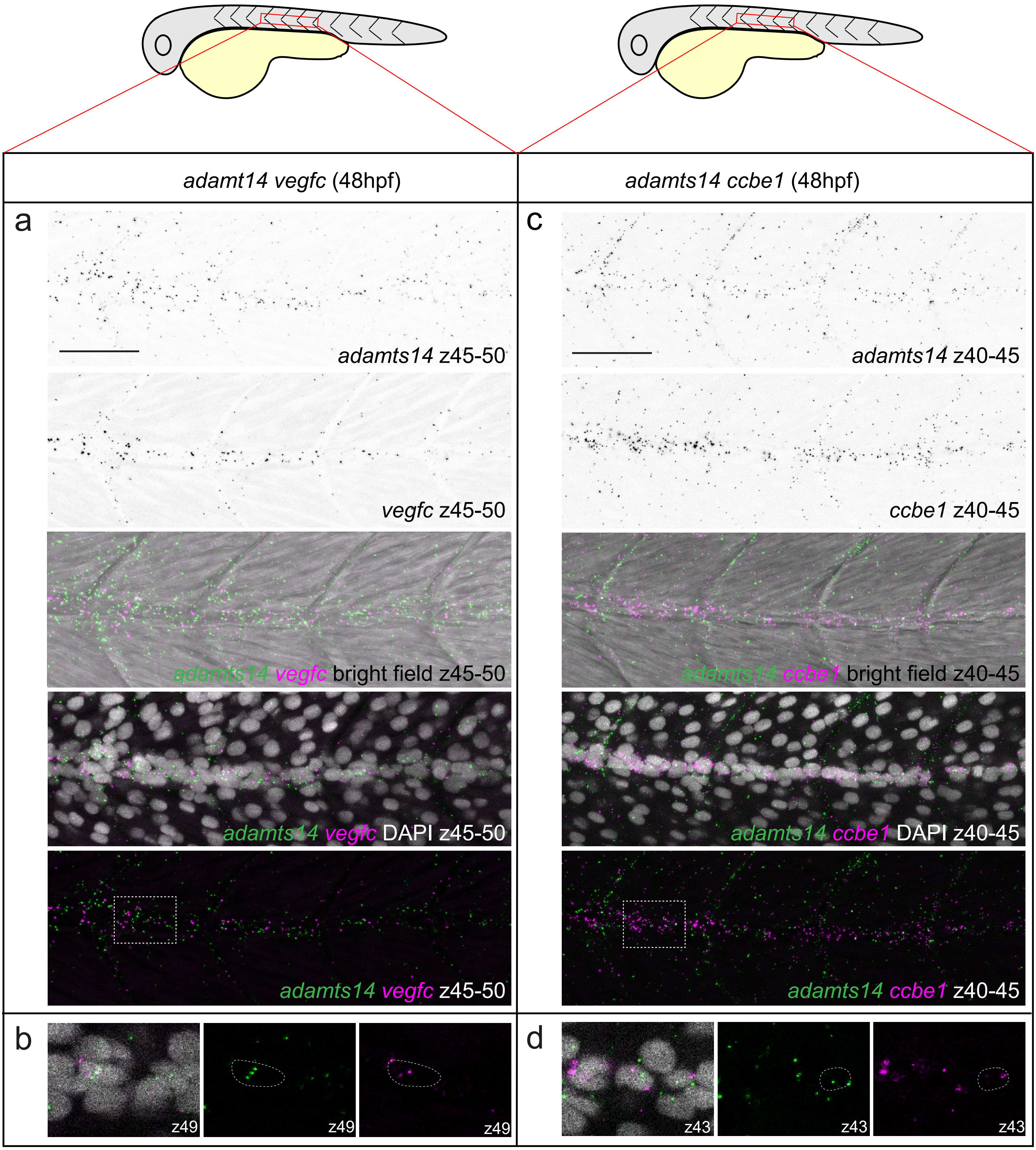

Supplementary Figure 7 Co-expression of *adamts14* with *vegfc* and *ccbe1* mRNA in cells at the HM.

Simultaneous detection of mRNA granules for *adamts14* and *vegfc* (a, b) and *adamts14* and *ccbe1* (c, d) by RNAscope in 48hpf embryos. Overview pictures (a, c) represent partial z-projections of the indicated ranges and show the overall mRNA distribution for the respective genes as well as the tight packing of nuclei (DAPI staining) in the HM area. Co-expression analysis of the individual transcript combinations was performed on single confocal sections (b, d) focusing exclusively on RNA granules that reside within the same nucleus (indicated by DAPI staining or outlined by a dotted circle). Scale bars: 50µm. hpf: hours post fertilization, HM: horizontal myoseptum.

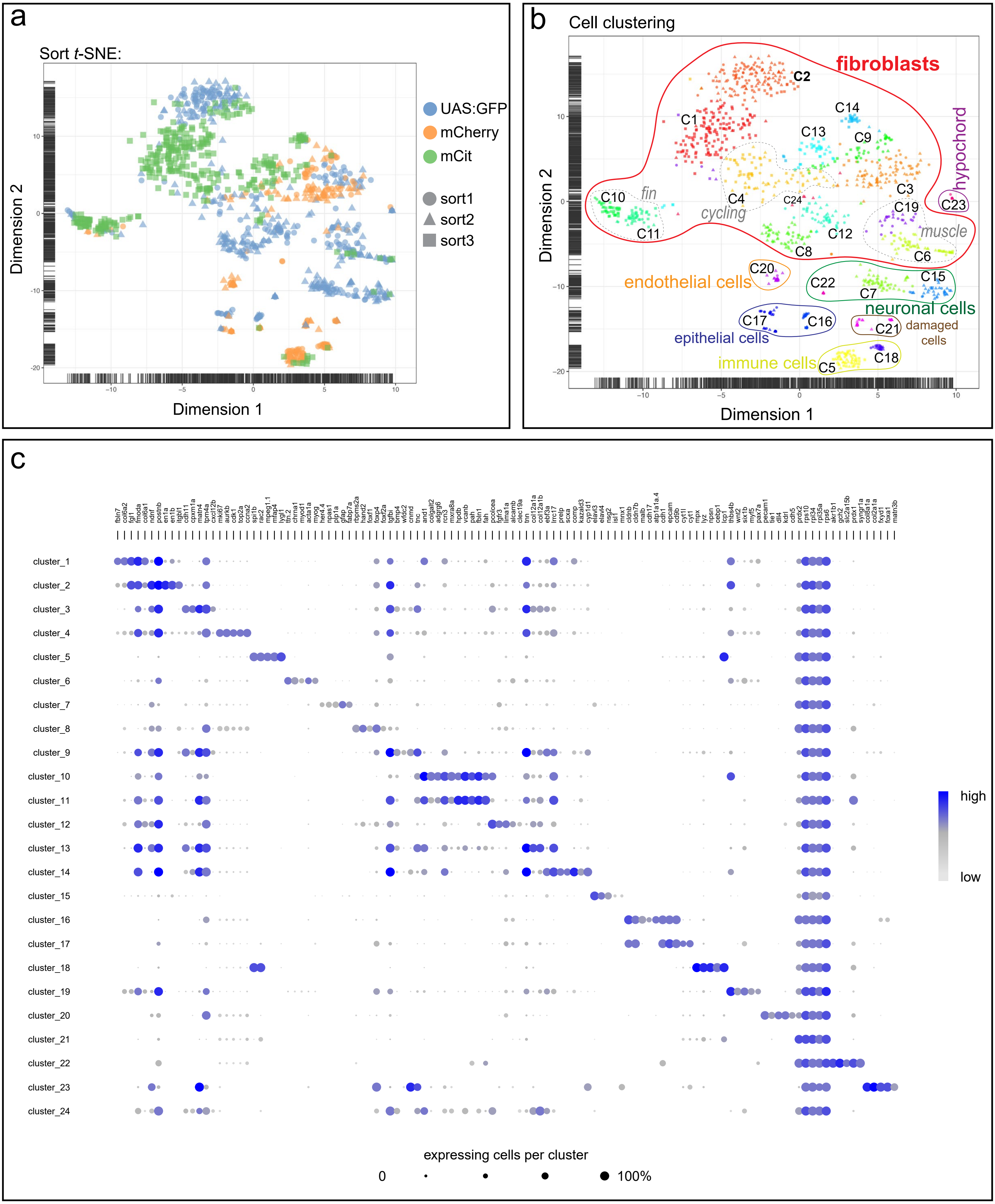

Supplementary Figure 8 Annotation of Pagoda2 cell clusters based on marker gene expression.

a) t-SNE plot indicating the distribution of cells amongst the clusters that were originally sorted for expression of *vegfc:Gal4FF; UAS:GFP* (blue), *kdrl:mCherry* (orange) or *pdgfra:mCitrine* (green). b) t-SNE plot with annotation of clusters. Note that cells with a general fibroblast signature are located within the upper half of the plot, encircled by a red line. c) Dot plot indicating the expression levels of selected marker genes within the individual clusters that were used for the annotation. The size of the dot indicates the number of cells expressing a particular transcript while the color gradient indicates the mean expression value per cluster.

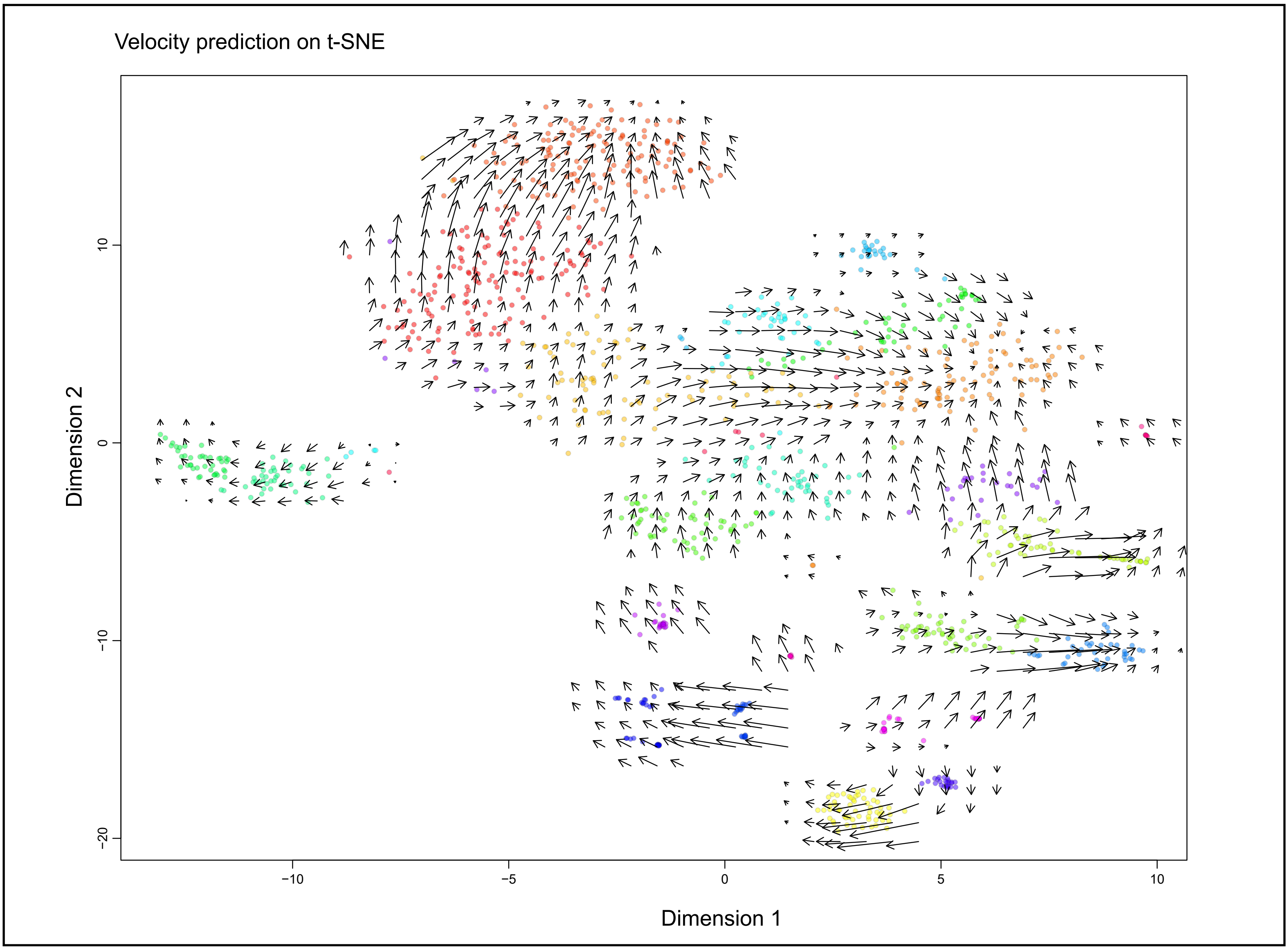

Supplementary Figure 9 Velocity plot in the t-SNE space.

The velocity plot indicates the predicted differentiation path (indicated by arrows) of the individual cells based on spliced vs. un-spliced RNA transcripts information.

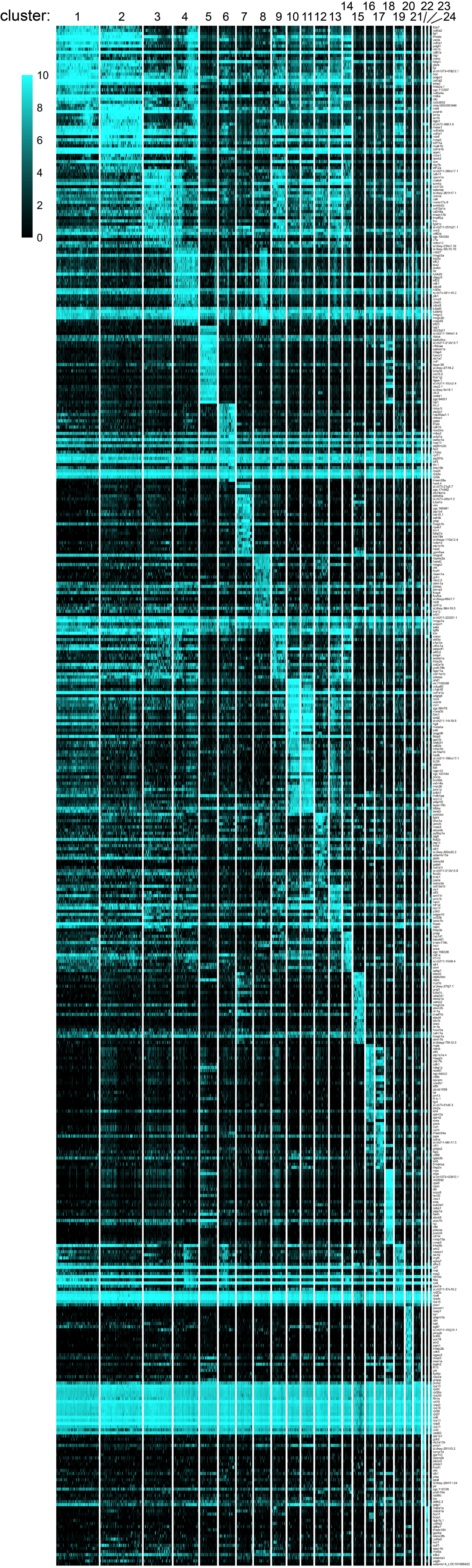
cluster:
1
2
3
4
5
6
7
8
9
10
11
12
13
14
15
16
17
18
19
20
21
22
23
24
10
8
6
4
2
0

Supplementary Figure 10 Heatmap indicating the relative expression of transcripts most specific for each pagoda2 cell cluster.

Depicted is the relative expression (blue=high; black=low) of the indicated transcripts in each of the pagoda2 cell clusters.

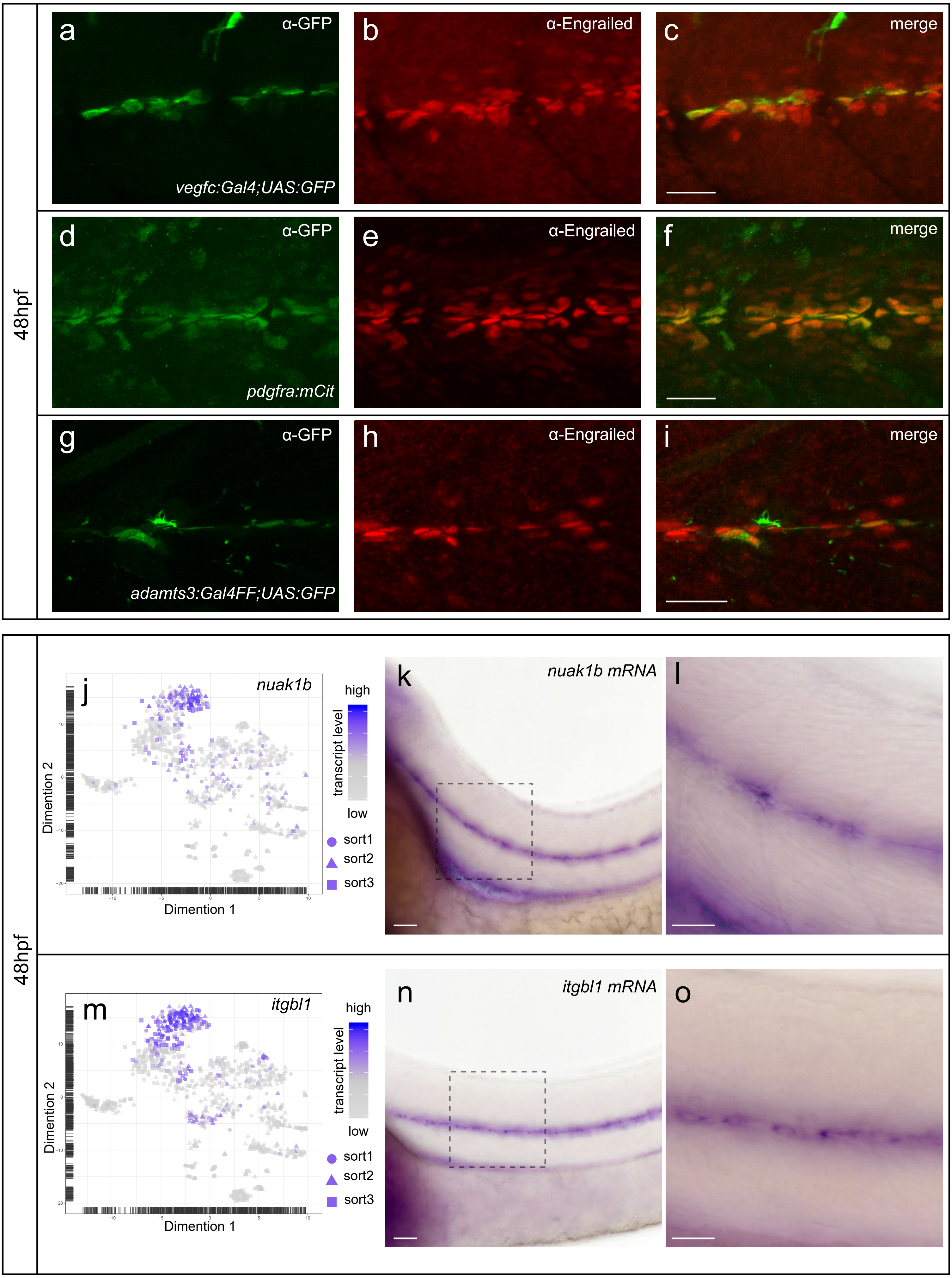
48hpf
a
α-GFP
vegfc:Gal4;UAS:GFP
b
α-Engrailed
c
merge
d
α-GFP
pdgfra:mCit
e
α-Engrailed
f
merge
g
α-GFP
adamts3:Gal4FF;UAS:GFP
h
α-Engrailed
i
merge
48hpf
j
nuak1b
Dimention 2
Dimention 1
high
transcript level
low
sort1
sort2
sort3
k
nuak1b mRNA
l
m
itgbl1
Dimention 2
Dimention 1
high
transcript level
low
sort1
sort2
sort3
n
itgbl1 mRNA
o

Supplementary Figure 11 Fibroblasts at the horizontal myoseptum express Engrailed proteins.

a-i) HM region of 48hpf embryos that were stained with anti-Engrailed (red) and anti-GFP (green) antibodies. a-c) All cells expressing the *vegfc:Gal4FF; UAS: GFP* reporter at the HM do co-express Engrailed proteins. d-f) *pdgfra:mCitrine* positive fibroblasts at the HM are also positive for Engrailed. g-i) Partial z-projections of the HM region reveal a co-expression of the *adamts3* reporter and Engrailed proteins within fibroblasts. j, m) Two additional examples of genes whose transcripts are highly enriched within fibroblast cluster 2 are *nuak1b* and *itgbl1* (see Supplementary Tables 1-3). The mRNA of both genes (k, l, n, o) can be detected specifically at the midline by ISH at 48hpf, thereby validating the notion that the cells in cluster 2 represent the fibroblast subpopulation located at the HM. Scale bars in a-i: 25 µm; k, l, n and o: 50 µm. hpf: hours post fertilization, HM: horizontal myoseptum.

Supplementary Table 1. Differential gene expression analysis of cells in cluster 2 compared to all other cells

gene symbol	log2-fold change
ndnfl	6,07
si:ch211-269c21,2	4,72
sned1	4,47
en1a	4,39
lamb2	3,92
en1b	3,70
nuak1b	3,68
rbp7b	3,67
pappab	3,65
itgbl1	3,58
zmp:0000000846	3,57
ENSDARG00000097771	3,57
zgc:162612	3,53
ccbe1	3,52
glt8d2	3,31
enpp2	3,09
hhip	3,02
hic1	2,96
anos1b	2,83
si:ch73-366l1.5	2,79
klf12a	2,73
pbx1a	2,62
foxd2	2,60
loxa	2,58
ENSDARG00000025320	2,55
ndnf	2,54
kitlga	2,54
cyyr1	2,51
palld	2,51
cxcl12a	2,49
apln	2,48
calcrla	2,44
htra1a	2,42
tcf7l1a	2,40
fgl1	2,39
znf469	2,30
apcdd1l	2,23
stk17a	2,22
meox1	2,22
angptl7	2,22

Listed are the 40 most enriched transcripts within scRNAseq cluster 2 when compared to all other clusters.

Supplementary Table 2. Differential gene expression analysis of cluster 2 compared to all other fibroblast clusters

gene symbol	log2-fold change
ndnfl	6,21
si:ch211-269c21.2	4,41
sned1	4,22
en1a	3,98
lamb2	3,50
en1b	3,45
nuak1b	3,34
pappab	3,25
rbp7b	3,24
itgbl1	3,16
ENSDARG00000097771	3,15
zmp:0000000846	3,15
ccbe1	3,10
zgc:162612	3,09
glt8d2	2,89
tox	2,85
apln	2,81
hic1	2,71
hhip	2,69
enpp2	2,65
si:ch73-366l1.5	2,58
pbx1a	2,52
anos1b	2,43
tshz3b	2,40
klf12a	2,36
foxd2	2,23
ndnf	2,22
cyyr1	2,22
tcf7l1a	2,20
palld	2,20
kitlga	2,19
calcrla	2,14
ENSDARG00000025320	2,12
loxa	2,11
cxcl12a	2,07
dchs1b	2,04

Shown are all transcripts with an enrichment higher than 2 (log2-fold change) within the scRNAseq cluster 2 when compared to all other fibroblast clusters.

Supplementary Table 3. Differential gene expression analysis of cluster 2 compared to the most closely related cluster 1

gene symbol	log2-fold change
sned1	6,22
fat4	5,45
ndnfl	5,24
ebf1a	4,44
glt8d2	4,26
agtr2	4,25
tnc	4,02
zgc:162612	3,90
si:ch211-251b21.1	3,82
foxc1a	3,81
lamb2	3,80
foxd2	3,76
apln	3,56
hic1	3,49
kitlga	3,35
palld	3,34
itga5	3,29
tox	3,29
pcolcea	3,28
nuak1b	3,22
zgc:154093	3,22
si:ch211-269c21.2	3,21
cyp2ad3	2,97
pappab	2,97
ednraa	2,81
fibina	2,79
zmp:0000000846	2,79
enc1	2,78
ndnf	2,70
ccbe1	2,69
si:ch211-286o17.1	2,69
sema3fa	2,59
rbp7b	2,57
en1a	2,45
serpine1	2,44
si:ch73-366l1.5	2,40
tcf7l1a	2,40
iqsec1b	2,39
tshz3b	2,35
pbx1a	2,27

Shown are the 40 most enriched transcripts in scRNAseq cluster 2 in comparison to the presumptive progenitor population in cluster 1.

Supplementary Table 4. Number of cells that express or co-express *adamts3*, *adamts14*, *ccbe1* and *vegfc* in each cluster

cluster	total	*adamts3*	*adamts14*	*vegfc*	*ccbe1*	*vegfc & ccbe1*	*adamts3 & ccbe1*	*adamts3 & vegfc*	*adamts14 & ccbe1*	*adamts14 & vegfc*
1	141	16	6	14	18	10	2	2	1	0
2	137	13	4	59	99	47	11	5	2	2
3	93	8	1	14	18	3	1	1	0	0
4	82	14	2	14	29	10	2	2	1	0
5	56	0	0	1	0	0	0	0	0	0
6	54	2	0	3	0	0	0	0	0	0
7	51	19	1	5	0	0	0	3	0	0
8	54	4	0	9	2	0	0	1	0	0
9	45	0	2	0	8	0	0	0	1	0
10	40	10	0	0	0	0	0	0	0	0
11	40	5	0	0	0	0	0	0	0	0
12	43	5	0	4	7	0	1	0	0	0
13	38	5	0	1	12	0	2	1	0	0
14	28	1	0	0	5	0	0	0	0	0
15	36	0	0	1	3	1	0	0	0	0
16	26	0	0	0	1	0	0	0	0	0
17	28	0	1	2	0	0	0	0	0	0
18	24	0	0	0	0	0	0	0	0	0
19	30	2	0	2	0	0	0	0	0	0
20	21	1	1	3	0	0	0	0	0	0
21	24	0	0	0	0	0	0	0	0	0
22	6	0	0	0	0	0	0	0	0	0
23	6	0	0	4	0	0	0	0	0	0
24	6	2	0	1	0	0	0	0	0	0

Supplementary Table 5. Clone ID of BACs used to generate the indicated reporter lines

Tg(adamts3:Gal4FF)	DKEY-156D16
Tg(vegfc:Gal4FF)	DKEY-87F9
Tg(vegfc:mCit)	DKEY-87F9
Tg(ccbe1:mCit)	CH73-375H6
Tg(ccbe1:Gal4FF)	CH73-375H6
Tg(pdgfra:mCit)	CH211-93B20

Supplementary Table 6. Genotyping primers

	fw	rev
adamts2 exon6	GATTTGTGTTGCTGTTGTCAGTCCAT	GCAATGACATGAGGTTGGGTAAATAA
adamts2_like exon2	CGGAATTGCATTGTTATAGAAATCAGAAG	ATGCCTGTGACTGGAGCGTAACC
shh:vegfc	GCAGTTGCGTTCAGCGGGTAGTGT	GCTGATGTATGAAGTGCTGATGTT

Supplementary Table 7. KASPar genotyping primers

	wild-type	mutant	common
adamts3^{hu11198} exon5	GAAGGTGACCAAGTTCATGCTCAGATAGTTCTGCACATGCTCCTTCC	GAAGGTCGGAGTCAACGGATTCAGATAGTTCTGCACATGCTCCTTCa	GAGACATGCTGGTGATGAGGACTACAAC (common forward primer)
adamts3^{hu10891} exon3	GAAGGTGACCAAGTTCATGCTGCCATGGTGGAATGGCACGATG	GAAGGTCGGAGTCAACGGATTGCCATGGTGGAATGGCACGATc	TTCTGTTATCAGTCGCATTTCCAGC (common reverse primer)
adamts14^{hu11304} exon3	GAAGGTGACCAAGTTCATGCTTGGGAACAAACATCAACATTGTGCT	GAAGGTCGGAGTCAACGGATTTGGGAACAAACATCAACATTGTGCc	GCCTTACCTGTCGATATCCCACCA (common reverse primer)

Supplementary Table 8. Primers used for the generation of *adamts3* and *adamts14* ISH probes

adamts3_probe1-for	ATGGTTGTCCTGTCACTTAGGTTACTCATAG
adamts3_probe1-rev (T3 promoter)	cattaaccctcactaaagggaaGGAATCGGACGACAGAATCATCAAC
adamts3_probe2-for	TCCTGGGGCTAGACATGTGA
adamts3_probe2-rev (T3 promoter)	cattaaccctcactaaagggaaTGTGGTTGTGTACAGTCCTGT
adamts3_probe2-for	TCAAATCCGTACTGTGCGCT
adamts3_probe3-rev (T3 promoter)	cattaaccctcactaaagggaaGTTCCTCCTCTGTTTCCGCA
adamts14_probe1-for	ACTCGGTGGTTCGCTTTCAT
adamts14_probe1-rev (T3 promoter)	cattaaccctcactaaagggaaCGGGACCAGTGATATCGGTG
adamts14_probe2-for	ATGACCCGTGTAAGCAGCTG
adamts14_probe2-rev (T3 promoter)	cattaaccctcactaaagggaaACGGGTCTGTGTAACTGCAG
adamts14_probe2-for	GTTTGATATTCCTGCGGGCG
adamts14_probe3-rev (T3 promoter)	cattaaccctcactaaagggaaCTGTTGGTGTTGCAGCGTTT

Supplementary Table 9. RNAscope probe design

probe	sequence accession numbers	range in bp	channel
adamts14	XM_017358603.2	1697-2625	1
ccbe1	NM_001163923.1	208-1223	2
pdgfra	NM_131459.2	127-1039	3
vegfc	NM_205734.1	435-1486	1 and 2
