# Supplementary material for: Specific fibroblast subpopulations and neuronal structures provide local sources of Vegfc-processing components during zebrafish lymphangiogenesis
Source: Nat Commun. 2020 Jun 1;11:2724. doi: 10.1038/s41467-020-16552-7 (PMC7264274; doi:10.1038/s41467-020-16552-7)
Supplement: Supplementary file 11 — Reporting Summary [file 41467_2020_16552_MOESM11_ESM.pdf]

nature research

Corresponding author(s): Stefan Schulte-Merker
Andreas van Impel

Last updated by author(s): Apr 30, 2020

Reporting Summary

Nature Research wishes to improve the reproducibility of the work that we publish. This form provides structure for consistency and transparency in reporting. For further information on Nature Research policies, see Authors & Referees and the Editorial Policy Checklist .

Statistics

For all statistical analyses, confirm that the following items are present in the figure legend, table legend, main text, or Methods section.

n/a	Confirmed	
☐	☒	The exact sample size (*n*) for each experimental group/condition, given as a discrete number and unit of measurement
☐	☒	A statement on whether measurements were taken from distinct samples or whether the same sample was measured repeatedly
☐	☒	The statistical test(s) used AND whether they are one- or two-sided<br>*Only common tests should be described solely by name; describe more complex techniques in the Methods section.*
☒	☐	A description of all covariates tested
☒	☐	A description of any assumptions or corrections, such as tests of normality and adjustment for multiple comparisons
☐	☒	A full description of the statistical parameters including central tendency (e.g. means) or other basic estimates (e.g. regression coefficient) AND variation (e.g. standard deviation) or associated estimates of uncertainty (e.g. confidence intervals)
☒	☐	For null hypothesis testing, the test statistic (e.g. *F*, *t*, *r*) with confidence intervals, effect sizes, degrees of freedom and *P* value noted<br>*Give P values as exact values whenever suitable.*
☒	☐	For Bayesian analysis, information on the choice of priors and Markov chain Monte Carlo settings
☒	☐	For hierarchical and complex designs, identification of the appropriate level for tests and full reporting of outcomes
☒	☐	Estimates of effect sizes (e.g. Cohen's *d*, Pearson's *r*), indicating how they were calculated

Our web collection on statistics for biologists contains articles on many of the points above.

Software and code

Policy information about availability of computer code

Data collection	Leica LAS X 3.5.6.21594, Nikon NIS-Elements 4.40.00, FACSDiva 8.0.1
Data analysis	Adobe Photoshop 2017, Adobe Illustrator CC 2015, Adobe Illustrator CC 2015, Fiji [(Fiji Is Just) ImageJ2.0.0-rc-43/1.52u], GraphPad Prism 8.3.0, Bitplane Imaris 8.2.1, Illumina pipeline (bcl2fastq 2.19.0.316), TopHat 2.1.1 with Bowtie2 2.2.6, trim galore 0.4.4, samtools 1.9, featureCounts 1.4.6-p5, velocyto 0.17.17, velocyto.R 0.6, SingleCellExperiment 1.4.1, pathway and gene set overdispersion analysis (pagoda2) 0.1.0, FlowJo 10.6.1

For manuscripts utilizing custom algorithms or software that are central to the research but not yet described in published literature, software must be made available to editors/reviewers. We strongly encourage code deposition in a community repository (e.g. GitHub). See the Nature Research guidelines for submitting code & software for further information.

Data

Policy information about availability of data

All manuscripts must include a data availability statement. This statement should provide the following information, where applicable:

- Accession codes, unique identifiers, or web links for publicly available datasets
- A list of figures that have associated raw data
- A description of any restrictions on data availability

The authors declare that the data supporting the findings of this study are available within the paper and its supplementary information files. The raw single cell RNA sequencing data of this study have been deposited at NCBI's Gene Expression Omnibus with the accession number GSE146923 [https://www.ncbi.nlm.nih.gov/geo/query/acc.cgi?acc=GSE146923]. Source data underlying Figures 1k, 2j and 7b are provided as a Source Data file.

Field-specific reporting

Please select the one below that is the best fit for your research. If you are not sure, read the appropriate sections before making your selection.

☒ Life sciences ☐ Behavioural & social sciences ☐ Ecological, evolutionary & environmental sciences

For a reference copy of the document with all sections, see nature.com/documents/nr-reporting-summary-flat.pdf

Life sciences study design

All studies must disclose on these points even when the disclosure is negative.

Sample size	Sample sizes were not statistically predetermined but were chosen based on previous experience, standards in the field and previously published literature.
Data exclusions	no data were excluded
Replication	The following section indicates how often experiments have been repeated independently with similar results. The phenotypic analysis of adamts3 and adamts14 single and double mutants (Figure 1c-e, g-I, l-q, Supplementary Figure 1d, e) was performed at least five times, quantifications of PLs and venous ISVs (Figure 1k) were done at least 2 times and the analysis of all other single or double mutant combinations (Figure 1j and Supplementary Figure 1b, c) was done at least two times. The in vivo zebrafish Vegfc processing assays (Figure 2b-i) were done at least three times each while the in vitro processing assay (Figure 2j) was performed 5 times. The adamts3 ISH (Figure 3a-c) was done three times and the adamts3:Gal4FF transgene analysis was performed five times (Figure 3d, i-m) and two times in conjunction with the motoneuronal marker mnx1:GFP (Figure 3e-f). adamts14 expression analysis by ISH and RNAscope (Figure 4) was performed at least 3 times each. Cell transplantation assays for adamts3 (Figure 5) and adamts14 (Figure 6) were conducted at least 13 times each. Transgene co-expression (Figure 7c-p) was assessed three independent times and the expression pattern of the pdgfra reporter alone and in combination with other transgenes (Figure 8) was analyzed at least three times. Rescue experiments with a mature VEGFC construct (Figure 10) were performed six times. The axonal pattern of motoneurons in adamts3; adamts14 double mutants (Supplementary Figure 2) was analyzed at least two times for the indicated time points. Expression analysis for vegfc by ISH (Supplementary Figure 34) was performed once and the expression analysis for vegfc, ccbe1, adamts14 and pdgfra by RNAscope (Supplementary Figure 5-7) was done at least two times. The Engrailed antibody stainings have been performed twice for all combinations (Supplementary Figure 7k, l11a-ci) and the ISH against nuak1b (Supplementary Figure 7a-i11k, l) and itgbl1 (Supplementary Figure 7n, o11n, o) were done two times.
Randomization	Experimental groups were not pre-selected based on the genotype, hence experimental groups were randomized with regard to the genotype.
Blinding	Investigators were blinded to group allocation during data collection and quantification (genotyping of embryos was performed afterwards).

Reporting for specific materials, systems and methods

We require information from authors about some types of materials, experimental systems and methods used in many studies. Here, indicate whether each material, system or method listed is relevant to your study. If you are not sure if a list item applies to your research, read the appropriate section before selecting a response.

Materials & experimental systems

n/a	Involved in the study	
☐	☒	Antibodies
☐	☒	Eukaryotic cell lines
☒	☐	Palaeontology
☐	☒	Animals and other organisms
☒	☐	Human research participants
☒	☐	Clinical data

Methods

n/a	Involved in the study	
☒	☐	ChIP-seq
☐	☒	Flow cytometry
☒	☐	MRI-based neuroimaging

Antibodies

Antibodies used	mouse anti-engrailed 4D9 primary antibody (Developmental Studies Hybridoma Bank, University of Iowa, clone 4D9, Antibody Registry ID: AB_528224),<br>chicken anti-GFP primary antibody (Abcam, ab13970),<br>polyclonal goat anti-human VEGFC primary antibody (R&D, AF752),<br>sheep anti-Digoxigenin-AP Fab Fragments (Roche, cat-no. 11093274910, Lot: 16646822)<br>HRP-conjugated anti-mouse secondary antibody (Jackson Immuno Research),<br>Alexa 488-conjugated goat anti-chicken secondary antibody (Invitrogen, A11039)

Validation	The 4D9 anti-engrailed antibody has been frequently used for antibody stainings in zebrafish and was validated in Hatta et al., 1991.<br>The chicken anti-GFP antibody was used before (e.g. Karpanen et al., 2017) and the staining pattern was verified by comparison to the in vivo expression of the respective reporter lines.<br>The goat anti-human VEGFC antibody was used previously for Western Blot analysis, e.g. in Jah et al., 2017.<br>The anti-Digoxigenin antibody is a standard tool for in situ hybridization (see Schulte-Merker, 2002 or Thisse and Thisse, 2007).

Eukaryotic cell lines

Policy information about cell lines

Cell line source(s)	The human cell line HEK293-EBNA was bought from Invitrogen.
Authentication	The cell line was not authenticated.
Mycoplasma contamination	The employed cell lines tested negative for mycoplasma contamination.
Commonly misidentified lines<br>(See ICLAC register)	No commonly misidentified cell lines were used in the study.

Animals and other organisms

Policy information about studies involving animals; ARRIVE guidelines recommended for reporting animal research

Laboratory animals	The zebrafish (Danio rerio) was used as an animal model and males and females of the following mutant or transgenic lines have been employed: Tg(flt4:mCitrine)hu7135, Tg(flt1enh:tdTomato)hu5333, Tg(shh:vegfc-IRES-mTurquoise)hu10933, Tg(kdrl:HRASmCherry)s916, Tg(mnx1:GFP)ml2, Tg(NBT:dsRed)zf148, Tg(isl1:Gal4FF)hu6635, Tg(UAS:GFP)nkuasgfp1a, Tg(UAS:RFP)nkuasrfp1a, Tg(adamts3:Gal4FF)mu400, Tg(pdgfra:mCitrine, cmlc2:mTurquoise)mu401, Tg(vegfc:Gal4FF)mu402, Tg(vegfc:mCitrine)mu403 , Tg(ccbe1:mCitrine)hu6741, Tg(ccbe1:Gal4FF)hu8876, adamts3(hu10891), adamts3(hu11981), adamts14(hu11304), adamts2(hu11300), adamts2_like(hu11325). The individual lines have been outcrossed as much as possible to TL, AB or WIK strains. All analyzed embryos have been derived from matings of fish that were younger than 2 years.
Wild animals	the study did not involve wild animals
Field-collected samples	the study did not involve samples collected from the field
Ethics oversight	Animal experiments have been performed according to guidelines of the animal ethics committees at the University of Münster, Germany and the LANUV (Landesamt für Natur, Umwelt und Verbraucherschutz Nordrhein-Westfalen).

Note that full information on the approval of the study protocol must also be provided in the manuscript.

Flow Cytometry

Plots

Confirm that:

- [x] The axis labels state the marker and fluorochrome used (e.g. CD4-FITC).
- [x] The axis scales are clearly visible. Include numbers along axes only for bottom left plot of group (a 'group' is an analysis of identical markers).
- [x] All plots are contour plots with outliers or pseudocolor plots.
- [x] A numerical value for number of cells or percentage (with statistics) is provided.

Methodology

Sample preparation	Approximately 100 trunks of transgenic embryos at 48hpf were partially dissociated with 0.5% trypsin-EDTA (Gibco) and 10U DNaseI (Promega) in a total volume of 1ml for 30min at 30°C.
Instrument	FACSAria IIIu cell sorter (BD Biosciences)
Software	FACSDiva 8.0.1, FlowJo 10.6.1
Cell population abundance	Cells were used for subsequent single cell sequencing analysis, hence purity of the sorted cell population was not assessed.
Gating strategy	Gates have been set for each transgenic line based on a comparison with age-matched transgene-negative controls to exclude autofluorecent cells.

- [x] Tick this box to confirm that a figure exemplifying the gating strategy is provided in the Supplementary Information.
