# Supplementary material for: Specific fibroblast subpopulations and neuronal structures provide local sources of Vegfc-processing components during zebrafish lymphangiogenesis
Source: Nat Commun. 2020 Jun 1;11:2724. doi: 10.1038/s41467-020-16552-7 (PMC7264274; doi:10.1038/s41467-020-16552-7)
Supplement: Supplementary file 12 — Description of Additional Supplementary Files [file 41467_2020_16552_MOESM12_ESM.pdf]

Title: Supplementary Movie 1
Description: Venous sprouting and migration in wild-type embryo. Over-night imaging of a wild type embryo starting at 32hpf showing the sprouting and migration of venous endothelial from the PCV. Venous and lymphatic endothelial cells were labeled with flt4:mCit.

Title: Supplementary Movie 2
Description: Venous sprouting is blocked in adamts3; adamts14 double mutant embryos. Over-night imaging of an adamts3; adamts14 double mutant in which venous and lymphatic endothelial cells were labeled with flt4:mCit, starting at 32hpf.

Title: Supplementary Movie 3
Description: 3D-reconstruction and surface rendering depicting the expression of the adamts3 reporter line at the horizontal myoseptum at 48hpf. In the adamts3:Gal4FF; UAS:GFP; kdrl:mCherry double transgenic embryo adamts3-expressing cells are highlighted in green and endothelial cells in red.

Title: Supplementary Movie 4
Description: 3D-reconstruction and surface rendering of adamts3+ cell transplantations giving rise to motoneurons. Depicted is a rescue of vISV and PL formation in an adamts3; adamts14 double mutant at48hpf. Transplanted adamts3-expressing cells are shown in green, all transplanted cells are labeled in blue (dextran-Alexa647); flt4:mCit-positive endothelial cells are highlighted in yellow (or red in the surface rendering).

Title: Supplementary Movie 5
Description: 3D reconstruction and surface rendering of a transplantation giving rise to adamts3-expressing mesenchymal cells. Shown is a region exhibiting a rescue of vISV and PL development in an adamts3; adamts14 double mutant at48hpf. adamts3-expressing cells are shown in green and flt4:mCit positive endothelial cells are highlighted in yellow (or red in the surface rendering).

Title: Supplementary Movie 6
Description: 3D reconstruction and surface rendering of floorplate and PL in the transplantation of adamts14-expressing cells. The movie showed the rescue region of adamts3; adamts14 double mutant at48hpf. Dextran-Alexa647 labeled floor plate were in grey; flt4:mCit positive cells were in green; flt1:tdTom positive cells were in red.

Title: Supplementary Movie 7
Description: 3D reconstruction and surface rendering of mesenchymal cells and PL in the transplantation of adamts14-expressing cells. The movie showed the rescue region of adamts3; adamts14 double mutant at48hpf. Dextran-Alexa647 labeled transplanted cells were in grey; flt4:mCit positive cells were in green; flt1:tdTom positive cells were in red. In order to highlight the cells at segment boundary and horizontal myoseptum, surface rendering was applied.

Title: Supplementary Movie 8
Description: 3D reconstruction and surface rendering of mesenchymal cells at HM and PL in the transplantation of adamts14-expressing cells. The movie showed the rescue region of adamts3; adamts14 double mutant at48hpf. Dextran-Alexa647 labeled transplanted cells were in grey;

flt4:mCit positive cells were in green; flt1:tdTom positive cells were in red. In order to highlight the cells at the horizontal myoseptum, surface rendering was applied
